# Supplementary material for: Antibiofilm Activity on Candida albicans and Mechanism of Action on Biomembrane Models of the Antimicrobial Peptide Ctn[15–34]
Source: Int J Mol Sci. 2020 Nov 6;21(21):8339. doi: 10.3390/ijms21218339 (PMC7664368; doi:10.3390/ijms21218339)
Supplement: Supplementary file 1 [file ijms-21-08339-s001.pdf]

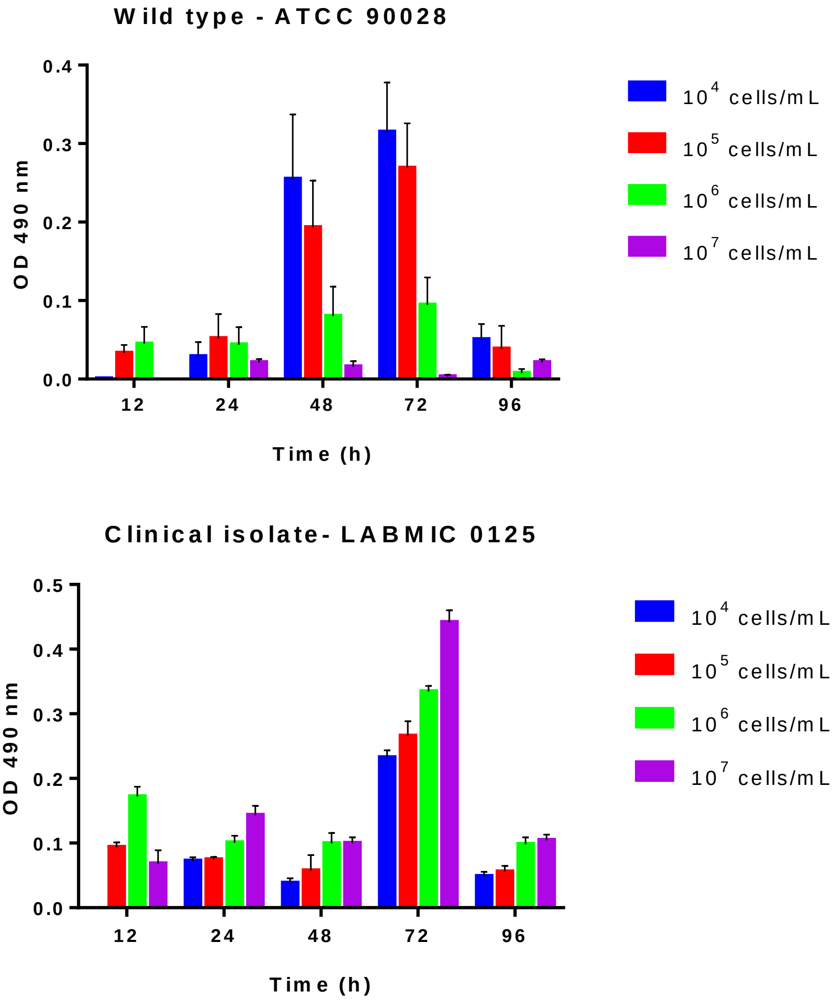

Figure S1. Colorimetric readings from XTT/menadione assays of biofilms formed by the two *C. albicans* strains. Each column represents the mean ± standard deviation (SD) from eight independent biofilm readings.

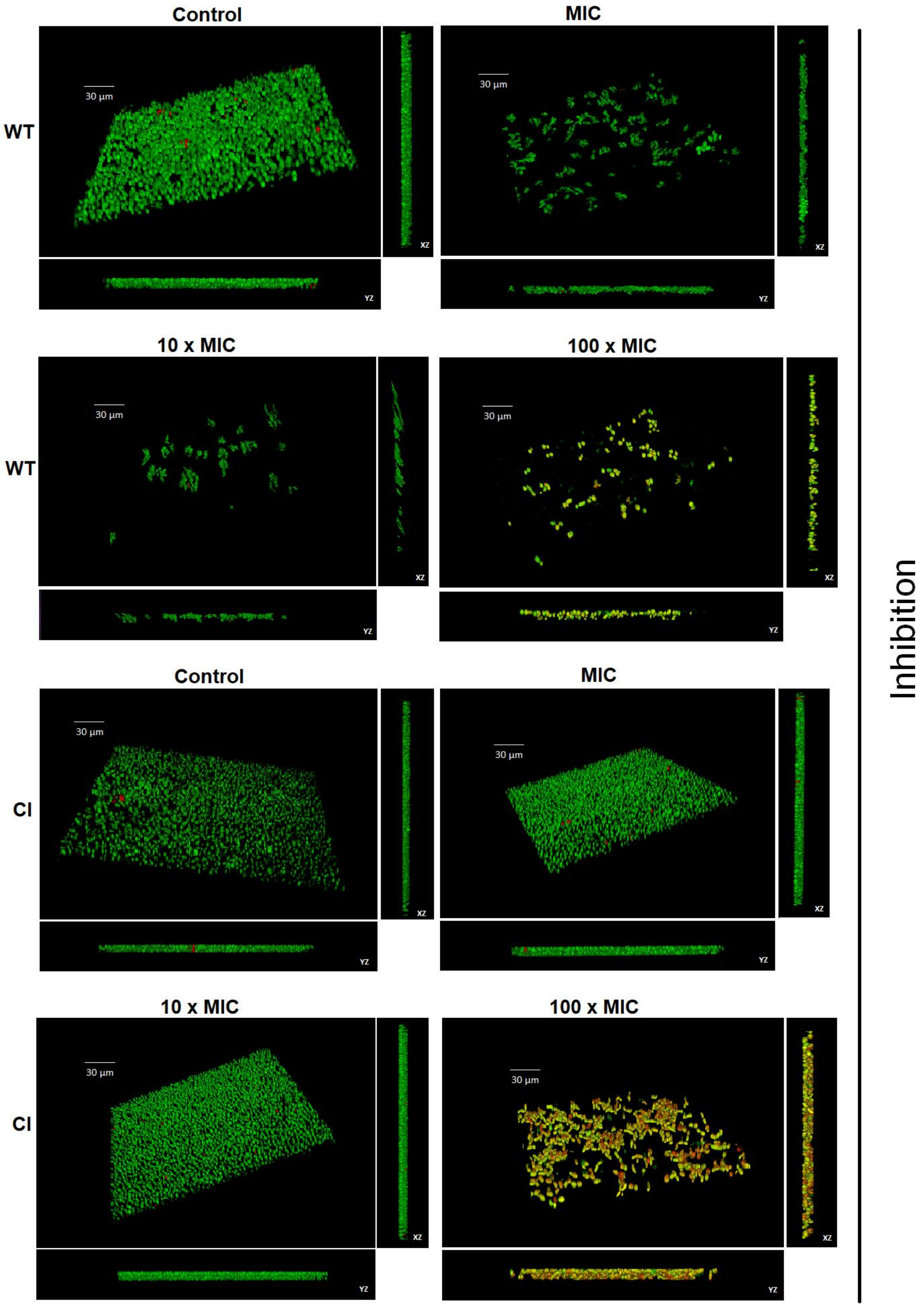

Figure S2 Effect of Ctn[15-34] on the inhibition of *C. albicans* biofilms. Z-tack images of the inhibition of *C. albicans* biofilms for wild type (WT) and clinical isolate (CI), in the absence (control) and presence of Ctn[15-34] at different concentrations. Images obtained with live/dead staining (SYTO®9 and PI, respectively) using 40-fold magnification. Images obtained using ImageJ software.

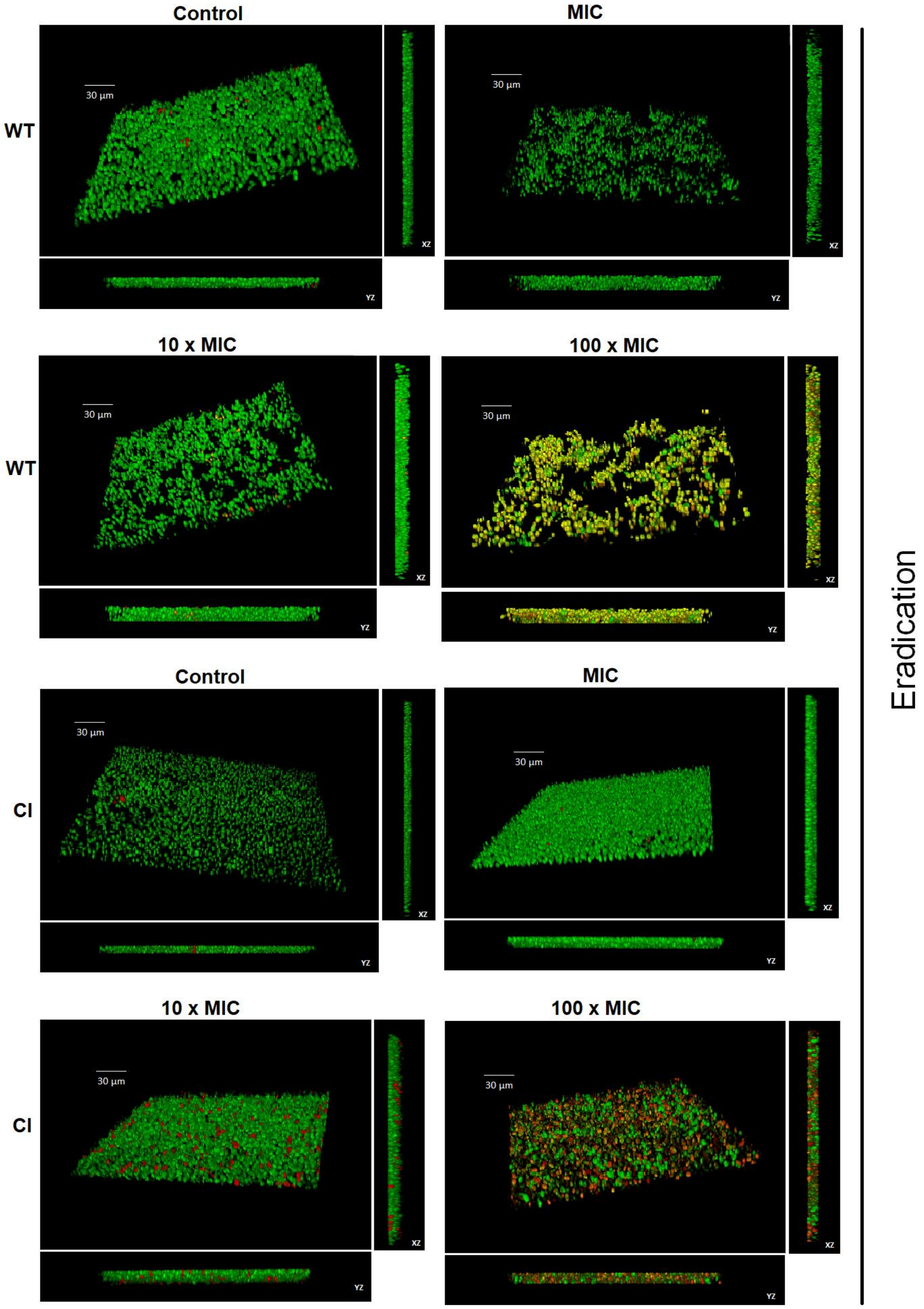

Figure S3 Effect of Ctn[15-34] on the eradication of *C. albicans* biofilms. Z-tack images of the eradication of *C. albicans* biofilms for wild type (WT) and clinical isolate (CI), in the absence (control) and presence of Ctn[15-34] at different concentrations. Images obtained with live/dead staining (SYTO 9 and PI, respectively) using 40-fold magnification. Images obtained using ImageJ software.
